# Endothelial Plaques as Sign of Hyphae Infiltration of Descemet's Membrane in Fungal Keratitis

**DOI:** 10.1155/2020/6083854

**Published:** 2020-05-26

**Authors:** Xiaolin Qi, Ting Liu, Man Du, Hua Gao

**Affiliations:** ^1^Eye Hospital of Shandong First Medical University, State Key Laboratory Cultivation Base, Shandong Provincial Key Laboratory of Ophthalmology, Shandong Eye Institute, Shandong First Medical University and Shandong Academy of Medical Sciences, Jinan, China; ^2^State Key Laboratory Cultivation Base, Shandong Provincial Key Laboratory of Ophthalmology, Shandong Eye Institute, Shandong First Medical University and Shandong Academy of Medical Sciences, Qingdao, China

## Abstract

**Background:**

To evaluate the relationship between corneal endothelial plaques and fungal hyphae infiltration in fungal keratitis.

**Methods:**

Retrospective cross-sectional study of 60 fungal keratitis patients who underwent keratoplasty between January 2013 and March 2017. The endothelial plaques were graded as follows: grade 1, 1–3 endothelial plaques; grade 2, 4–8 endothelial plaques; and grade 3, more than 8 endothelial plaques or dense, merging endothelial plaques. The fungal pathogen culture and histopathology of diseased Descemet's membrane were evaluated.

**Results:**

According to endothelial plaque grading, 3 patients were grade 1, 29 patients were grade 2, and 28 patients were grade 3. The PK surgery was performed in 57 patients with endothelial plaques of grade 2 and grade 3 and DALK surgery in 3 patients of grade 1. The predominating fungal pathogens were *Aspergillus* species (63.2%). All 57 patients with grade 2 and grade 3 had fungal hyphae in Descemet's membrane based on calcofluor white staining or PAS staining. In patients with grade 3, more hyphae and inflammatory cells were found in Descemet's membrane. The immunohistochemical staining of endothelial plaques revealed that CD15 and CD68 were positive in most cells. During the follow-up, 2 out of 3 patients who underwent DALK had recurrent fungal keratitis.

**Conclusions:**

Endothelial plaques are considered as a sign of hyphae infiltrating Descemet's membrane. PK should be performed once plaques are detected in endothelium during the surgery.

## 1. Introduction

Fungal keratitis (FK) is a severe infectious corneal disease in developing countries [1–3]. In China, more than 50% of infectious keratitis cases are the result of a fungal infection [4]. Clinical manifestations of fungal keratitis include elevated lesions and necrosis, pseudopodia, corneal ring, endothelial plaque, and hypopyon [3, 5]. According to the reported literature, the presence of endothelial plaque was considered as a risk factor for lamellar keratoplasty treatment failure [6–8]. However, due to the lack of histopathological evidence, the formation of endothelial plaques is related to anterior chamber reaction of severe fungal infections, or hyphae infiltration of Descemet's membrane remains unclear. Furthermore, it is often difficult to choose deep anterior lamellar keratoplasty (DALK) or penetrating keratoplasty (PK) when encountering endothelial plaques during keratoplasty surgery. In this study, we attempted to use histological evidence to show that endothelial plaques are a reliable sign of hyphae infiltration of Descemet's membrane, thus providing surgical guidance in these circumstances.

## 2. Methods

### 2.1. Patients

We adhered to the principles outlined in the Declaration of Helsinki, and this study was approved by the ethics committee of Shandong Eye Hospital. A total number of 242 patients with fungal keratitis underwent keratoplasty between January 2013 and March 2017, including DALK for 89 patients, and PK for 153 patients were reviewed retrospectively. The inclusion criteria were as follows: (1) the hyphae were detected by corneal smear examination or laser scanning confocal microscopy (Heidelberg Instruments, GmbH, Heidelberg, Germany); (2) over 4/5 of the corneal thickness was infected or infiltrated as observed by slit-lamp microscopy, laser scanning confocal microscopy, and anterior segment optical coherence tomography (As-OCT); (3) antifungal medication as reported in our previous studies [9, 10] was given for at least 2 weeks but was ineffective. The patients detected with no endothelial plaque and diagnosed with perforation were excluded from this study. Finally, a total of 60 patients (60 eyes) were included (26 men and 34 women). Their mean age was 40.5 years (range 31–68 years).

A comprehensive eye examination was performed with a slit-lamp, including measuring the size of fungal ulcer and the depth of hypopyon. The methods were as follows. Photos of the corneas were obtained with a digital camera at the slit-lamp (Topcon, DC-3), and a picture of a graduated scale under the same magnification ratio was taken. Then, the pictures of the corneas and the graduated scale were opened in Adobe Photoshop software. After dragging the graduated scale to the cornea with the move tool, the size of fungal ulcer and the depth of hypopyon were measured and recorded.

### 2.2. Endothelial Plaque Evaluation

All the surgeries were planned as DALK preoperatively, and the decision of performing DALK or PK was made according to the evaluation of endothelial plaques after exposure of Descemet's membrane with the big-bubble technique. All surgeries were performed by a single surgeon (H.G.). The detailed surgical procedure was introduced in our previous report [11]. After Descemet's membrane was exposed, the endothelial plaques were assessed under the surgical microscope and graded as follows: grade 1, 1–3 endothelial plaques; grade 2, 4–8 endothelial plaques; and grade 3, more than 8 endothelial plaques or dense, merging endothelial plaques. If only 1–3 endothelial plaques (grade 1) were visible, DALK was performed. If more than 3 endothelial plaques (grade 2-3) were visible, PK was performed instead. After endothelial plaque evaluation, patients with endothelial plaques of grade 1 continued the surgery as DALK, and those with grades 2 and 3 were converted to PK.

After surgery, the diseased Descemet's membrane and the corneal lamellar tissue were sent for fungal pathogen culture and histopathological examination with calcofluor white and periodic acid-Schiff (PAS) staining.

### 2.3. Calcofluor White Staining of Descemet's Membrane

After PK, Descemet's membranes were stained with calcofluor white staining. Briefly, a drop of 1% calcofluor white (Sigma, St. Louis, USA) was added to Descemet's membranes obtained during PK, which were incubated for 2 min at room temperature and washed three times in normal saline. Fungal hyphae were observed using a fluorescence E800 microscope (Nikon, Tokyo, Japan). With calcofluor white staining, the fungal hyphae were bright blue against a dark background.

### 2.4. Histopathological Examination and Immunohistochemical Detection

Corneal buttons obtained during DALK or PK surgery were fixed in 4% formalin. These corneal buttons were half-cut along the central line. Serially graded ethanol baths followed by xylene were used to dehydrate the tissues before they were immersed in paraffin wax. The samples were embedded in paraffin molds, and serial slices (4 *μ*m) were stained with PAS stain (Maxin, Fujian, China). The hyphae were observed by light microscopy (Olympus BX60, Tokyo, Japan). Six microscopic fields (×400) were randomly chosen in each cornea, and the number of fungal hyphae and inflammatory cells per field were counted for the statistical analysis.

The expressions of a neutrophil marker (CD15) and a macrophage marker (CD68) were detected with immunohistochemical staining. Briefly, 4 *μ*m-sections were obtained from the paraffin-embedded corneal buttons. The antigens were recovered by microwaving the sections, and the endogenous peroxidase activity was quenched with a 3% solution of hydrogen peroxide for 10 min. The sections were incubated with primary antibodies (mouse anti-CD15 or CD68; Maxin, Fujian, China) for 60 min at 37°C after which they were incubated with HRP-conjugated goat anti-mouse IgG for 30 min at 37°C. The peroxidase activity was visualized by incubating the sections in a solution of diaminobenzidine (DAB; Maxin, Fujian, China). Negative controls were performed in the absence of primary antibodies. Finally, the samples were mounted and examined under a microscope (Olympus BX60, Tokyo, Japan).

### 2.5. Statistical Analysis

All data were analyzed with SPSS software (version 17.0, SPSS Inc., Chicago, IL, USA). Student's *t* test was used to compare the hyphae and inflammatory cell counts between patients with grade 2 and grade 3 endothelial plaques. The data are shown as the mean ± standard deviation, and a *P* value <0.05 was considered statistically significant.

## 3. Results

### 3.1. Patient Information

KOH smears and laser scanning confocal microscopy were hyphae positive in all 60 patients. The mean size of fungal ulcer was 6.9 ± 0.6 mm × 6.2 ± 0.5 mm before surgery. Hypopyon was present in 38 eyes, measuring 1.5 ± 0.9 mm (range: 0.2–4 mm).

### 3.2. Endothelial Plaque Evaluation and Surgery

**Figure 1:**
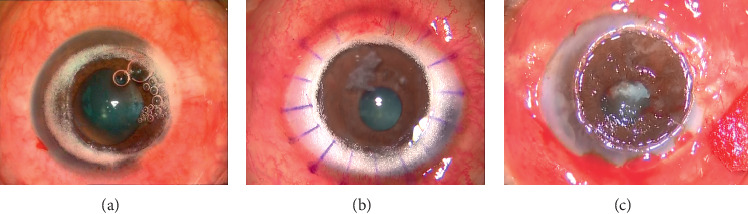
Representative pictures of grade 1 (a), grade 2 (b), and grade 3 (c) endothelial plaques after exposing Descemet's membrane during keratoplasty surgery.

All 60 patients had a varying degree of endothelial plaques, including 3 patients in grade 1, 29 patients in grade 2, and 28 patients in grade 3. The 3 patients with grade 1 underwent DALK surgery, whereas 57 patients with grade 2 and grade 3 underwent PK surgery (Figure 1). After the surgery, the mean follow-up time was 4.1 ± 2.6 months (range: 3–6 months).

### 3.3. Fungal Pathogen Distribution

In 38 fungal pathogens positively cultured, 24 (63.2%) were identified as *Aspergillus* species, 10 (26.3%) were *Fusarium* species, 2 (5.2%) were *Alternaria* species, and 2 (5.2%) were *Colletotrichum* species. There were also 22 unidentified species.

### 3.4. Calcofluor White Stain Evaluation

**Figure 2:**
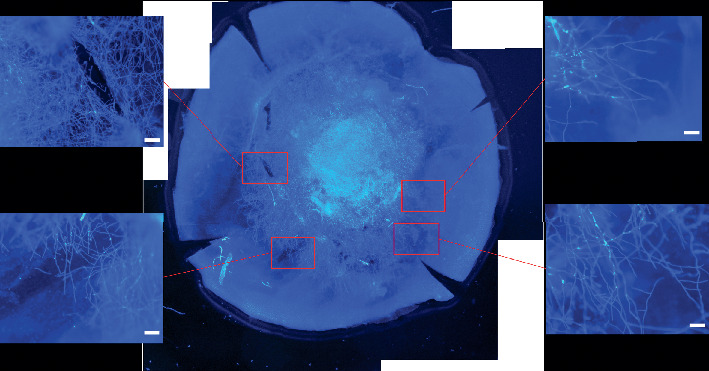
In a patient with grade 3 endothelial plaques, fungal hyphae were found in Descemet's membrane with calcofluor white stain by fluorescence microscopy (scale bar 20 *μ*m).

With calcofluor white stain, 29 patients with grade 2 and 28 patients with grade 3 were all hyphae positive in Descemet's membranes. The hyphae had many branches and were bamboo-structured and surrounded with inflammatory cells. The representative hyphae in Descemet's membranes of patients with grade 2 and grade 3 are shown in Figure 2.

### 3.5. Histopathology and Immunohistochemical Evaluation

**Figure 3:**
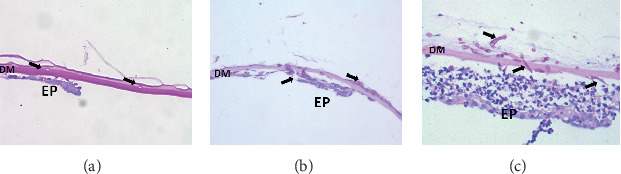
Hyphae (arrows) were found in patients with grade 1 (a), grade 2 (b), and grade 3 (c) with periodic acid-Schiff (PAS) stain. More hyphae and inflammatory cells were found in Descemet's membrane in patients with grade 3 (c) (scale bar 20 *μ*m).

**Figure 4:**
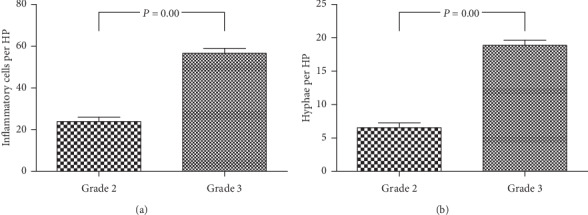
Significant differences were found between patients with grade 2 and grade 3 endothelial plaques (*P* < 0.01) including (a) the number of inflammatory cells and (b) hyphae present in Descemet's membrane per high-power field.

**Figure 5:**
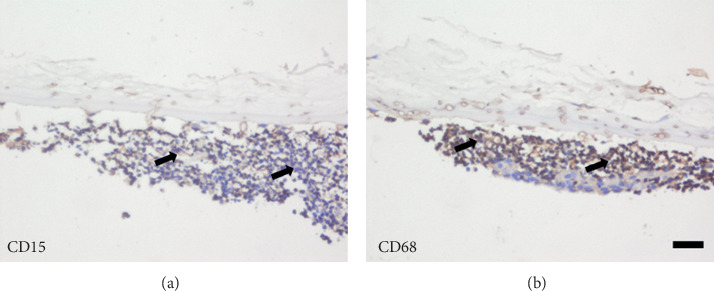
Immunohistochemical staining revealed CD15-positive neutrophils (brown color, arrowed in (a)) and CD68-positive macrophages (brown color, arrowed in (b) accumulated in the endothelial plaques (scale bar 20 *μ*m).

PAS staining the corneal stroma and Descemet's membrane was positive for fungal hyphae in all eyes. In patients with grade 3, the destruction of the cornea stroma was severe, and more hyphae and inflammatory cells were found in the deep stroma and Descemet's membrane. In patients with grade 2, the inflammation was moderate, and there were less hyphae in the deep stroma and Descemet's membrane (Figure 3). Both the number of hyphae and inflammatory cells per high-power field were significantly different between the patients with grade 3 and grade 2 (*P* < 0.01) (Figure 4). Immunohistochemical staining of endothelial plaques revealed that most cells were positive for CD15 (neutrophils) or CD68 (macrophages) (Figure 5).

### 3.6. Fungal Recurrence

Recurrent FK occurred in 2 out of 3 patients who underwent DALK. One eye recurred at 3 days after DALK surgery and at 5 days in another eye. The cultured fungal pathogens were both *Aspergillus* species in the 2 patients. No fungal recurrence was observed after PK surgery.

## 4. Discussion

The big-bubble-DALK was reported to be effective in the treatment of fungal keratitis. However, during the DALK surgery, if endothelial plaques were present, the doctor often faced a dilemma over whether to continue the DALK procedure or choose the PK procedure. There have been disputes about the possible correlation between hyphae infiltration and endothelial plaques [12–15]. Some researchers believed that the hyphae may invade the deep corneal stroma to cause an anterior chamber reaction as well as endothelial plaques with the progression of the disease, but no direct pathologic evidence was available [16, 17]. Therefore, it is necessary to study the components of corneal endothelial plaques and their relationship to hyphae penetrating Descemet's membrane, which may help clinical doctors to make an informed choice for surgical procedures.

Corneal endothelial plaques in FK contain a large number of acute inflammatory and immune cells. Our immunohistochemical staining revealed that the corneal endothelial plaques were mainly composed of neutrophils and macrophages surrounding the penetrating hyphae. This structure reflects the body's defense system against invading microorganisms such as the hyphae penetrating into the anterior chamber. Kiryu et al. [18] found that hyphae surrounded by neutrophils showed double or triple cell wall formation and sometimes a hypha-in-hypha structure in dexamethasone-treated corneal lesions. This special structure was regarded as a protective device for the survival of *Fusarium* species to evade the host's immune system.

Calcofluor white stain is an easy and direct staining method for fungi and is more sensitive for detecting fungal hyphae than the traditional PAS stain on paraffin sections. With direct calcofluor white stain, all patients had hyphae distribution in Descemet's membrane. Histological examination further verified that more hyphae and inflammatory cells were found in the grade 3 patients than in the grade 2 patients with PAS stain. Out of 3 patients with grade 1 endothelial plaques who underwent DALK, 2 patients had recurrent FK after DALK, further suggesting the close relationship between endothelial plaques and hyphae infiltration. These results indicate that endothelial plaques are a reliable sign of hyphae infiltration of Descemet's membrane.

The patients with 4/5 of the corneal thickness infected or infiltrated by hyphae and antifungal medication ineffective for 2 weeks are suggested to undergo keratoplasty. The exact procedure selection (DALK or PK) depends on the evaluation of deeper stroma and Descemet's membrane during the surgery. Surgeons can proceed with DALK when no hyphae and endothelial plaques are detected in the deeper stroma and Descemet's membrane. In a previous study by Dr. Gao [11], DALK was performed in a series of 23 patients with no hyphae in the posterior stroma near Descemet's membrane, and recurrence of FK was found in only two patients (8.7%), much lower than the reported values [13, 19, 20].

*Fusarium* is still the most common pathogen of fungal keratitis, followed by *Alternaria* and *Aspergillus* species, in Shandong Province, China [11, 21–23]. However, in this study, the postoperative fungal pathogen culture of the diseased Descemet's membrane showed that 24 (63.2%) out of the 38 positively cultured were identified as *Aspergillus* species. In addition, the cultured pathogens from the 2 patients with recurrent FK after DALK surgery were *Aspergillus* species. The main reason is that unlike *Fusarium* species with horizontally growing hyphae, most *Aspergillus* hyphae grow vertically in corneal stroma, so the deep stroma and Descemet's membrane are prone to be invaded. The rudimentary hyphae in the deep stroma and Descemet's membrane that are not cleared by DALK may cause fungal recurrence postoperatively. Hence, PK should be performed once plaques are detected in endothelium during the surgery, which means hyphae has penetrated Descemet's membrane to reduce the risk of fungal recurrence.

In conclusion, endothelial plaques are considered as a sign of hyphae infiltrating Descemet's membrane. The predominating fungal pathogens of diseased Descemet's membrane were *Aspergillus* species (63.2%). Once plaques are detected in endothelium during the surgery, PK should be performed to reduce the risk of fungal recurrence.

## Data Availability

All data and material are available within the article.

## Additional Points

Synopsis. Corneal endothelial plaques in FK contain a large number of bamboo-like hyphae and inflammatory cells. *Aspergillus* species is the predominating fungal pathogens capable of penetrating Descemet's membrane readily.
